# Harnessing Spinal Cord Stimulation and Neuromodulation for Functional Restoration: From Pain Management to Motor Recovery

**DOI:** 10.3390/brainsci16050476

**Published:** 2026-04-29

**Authors:** Wende Li, Xiaoyu Xia

**Affiliations:** Department of Neurosurgery, the Seventh Medical Center of Chinese PLA General Hospital, Beijing 100700, China; wenwendege@126.com

**Keywords:** spinal cord stimulation, neuromodulation, motor recovery, closed-loop systems, neuroplasticity

## Abstract

**Highlights:**

**What are the main findings?**

- Spinal cord stimulation (SCS) modulates spinal and supraspinal circuits involved in motor recovery and neuroplasticity.
- Advances in stimulation paradigms and closed-loop systems improve the precision and personalization of neuromodulation.

**What are the implications of the main findings?**

- SCS is evolving from a pain management therapy toward a circuit-based strategy for functional restoration.
- Integration with multimodal neuromodulation may enhance rehabilitation outcomes after spinal cord injury and stroke.

**Abstract:**

Spinal cord stimulation (SCS) has expanded beyond pain treatment, becoming a neuromodulatory method capable of recruiting spinal and supraspinal circuits involved in motor recovery. This review summarises mechanistic knowledge, supports engineering developments, and describes the changing clinical translation of SCS in rehabilitation. Mounting scientific data shows that SCS’s effects go beyond dorsal column modulation and may involve segmental networks that promote activity-dependent plasticity and sensorimotor pathway restoration, probably due to a combination of Hebbian and non-Hebbian mechanisms (synaptic potentiation, interneuronal reorganisation, and altered afferent–efferent coupling). More recent advances, such as bursts and the high-frequency paradigm, closed-loop control, and data-driven parameter optimisation methods, improve the precision, stability, and calibration of stimulation for each individual. By combining SCS with non-invasive forms of neuromodulation (TMS, tDCS, and peripheral nerve stimulation), one can potentially further intensify corticospinal plasticity and maintain improvements in functions. Spinal cord stimulation remains an established treatment for chronic neuropathic pain, including failed back surgery syndrome and complex regional pain syndrome. In recent years, however, increasing attention has been directed toward its potential role in motor recovery after spinal cord injury and stroke. Progress in this area is limited by patient heterogeneity, variability in outcome measures, the complexity of multimodal rehabilitation protocols, and regulatory and logistical constraints—particularly when adaptive or closed-loop systems are used. Current evidence suggests that motor-restorative applications of SCS should be interpreted cautiously and integrated within carefully designed rehabilitation programmes, with attention to patient selection and realistic expectations regarding the durability of the benefit.

## 1. Introduction

The use of SCS was initiated to relieve refractory neuropathic pain, supported by decades of clinical practice in failed back surgery syndrome and complex regional pain syndrome [1,2]. In the last ten years, advances at the interface of systems neuroscience, biomedical engineering, and neurorehabilitation have broadened this approach: SCS is now increasingly viewed as offering a neuromodulatory approach that can be used to manipulate spinal circuits to restore disrupted sensorimotor function [3,4]. This shift from symptomatic analgesia to specific circuit modulation is harmonised with a growing body of literature indicating that the damaged spinal cord has much plastic potential, even at chronic stages [5].

There is experimental and initial clinical evidence that epidural and transcutaneous stimulation has the potential to increase the excitability of intact descending pathways, induce rearrangement within propriospinal interneuronal networks, and improve sensorimotor integration, which could be utilised to stimulate voluntary motor performance in a few participants with a spinal cord injury or post-stroke hemiparesis [6,7]. These responses are probably a combination of Hebbian and non-Hebbian plasticity, which are dependent on the timing, frequency, and spatial targeting of the stimulations. At the same time, the paradigms of bursts, high frequency, closed-loop control, and machine-learning-assisted parameter optimisation have expanded the programmable design space of SCS. SCS is considered to have potential for use together with complementary treatments (e.g., TMS, tDCS, peripheral nerve stimulation, and robotic gait training) that may allow the multi-level control of cortical–spinal and peripheral nets [8].

To date, motor-restorative effects have only been reported in relatively small, highly controlled cohorts which, in many cases, require intensive therapist support, body-weight-assisted gait training, and multimodal rehab regimens [9]. The response to treatment is still nonhomogeneous based on the morphology of the lesions, chronicity, neuroinflammatory milieu, and interaction with behaviour. Methodological variability—especially in terms of electrode placement, stimulation paradigms, and outcome measures—limits cross-study comparability and synthesis. In addition to efficacy, real-world application is limited by the surgical risk, logistics, changing regulations, and health-system factors, such as cost and the training of clinicians.

With this promise of science and scope of translation, a rigorous synthesis is justified. This review synthesises mechanistic, engineering, and clinical evidence of SCS’s established analgesic uses and its future uses in motor recovery, the strength of the evidence, major limitations, and priorities for future clinical use and research.

### Literature Search and Review Strategy

This structured narrative review synthesises preclinical, translational, and clinical studies on spinal cord stimulation for motor restoration and pain indications. A literature search was conducted in PubMed, Web of Science, and Embase for studies published up to January 2026, using combinations of keywords including “spinal cord stimulation”, “motor recovery”, “epidural stimulation”, “transcutaneous stimulation”, “neuroplasticity”, and “closed-loop neuromodulation”.

No formal lower date limit was imposed. However, emphasis was placed on literature published within the past 10–15 years to reflect current technological and clinical developments, while earlier seminal mechanistic and clinical studies were included when essential for understanding the evolution of the field. Priority was given to peer-reviewed human studies, landmark mechanistic investigations, and translational research with clinical relevance. Case reports were included selectively when they represented field-defining observations. Studies unrelated to neuromodulation, spinal circuitry, or functional rehabilitation were excluded. Conference abstracts without full-text availability, duplicate publications, and studies lacking substantive methodological or clinical relevance were also excluded where applicable.

Titles and abstracts were screened by the authors for relevance to spinal circuit modulation, functional restoration, or established analgesic indications. Full texts of potentially eligible articles were then reviewed to confirm their suitability for inclusion. Study selection was guided by the objective of capturing representative and methodologically robust evidence across the translational spectrum, with particular emphasis on randomised controlled trials, prospective and well-characterised cohort studies, major clinical case series, and influential preclinical investigations. Where appropriate, reference lists of key articles were manually reviewed to identify additional relevant publications.

Given the heterogeneity of study designs and the rapidly evolving nature of motor-restorative applications, emphasis was placed on representative and high-impact studies rather than exhaustive enumeration. This approach enabled a structured synthesis of evidence across different levels of clinical maturity, from established analgesic indications to emerging and exploratory motor-restorative applications. This review aims to provide a critical and translational synthesis rather than a quantitative meta-analysis. Accordingly, it was not conducted as a formal systematic review and does not include quantitative pooling or a PRISMA-based selection framework. Instead, the methodology follows a structured narrative review approach designed to enhance transparency, reproducibility, and clinical interpretability while maintaining conceptual and translational integration across disciplines.

## 2. Mechanistic Foundations of Motor-Restorative Spinal Cord Stimulation

### 2.1. Sensory–Motor Gating and Engagement of Residual Pathways

**Figure 1:**
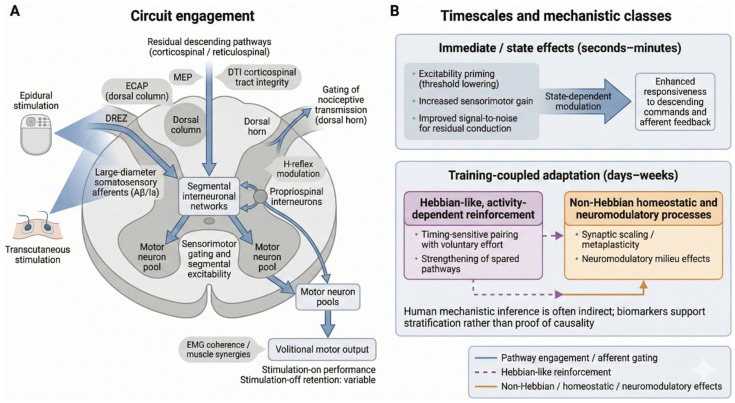
Mechanistic routes from spinal cord stimulation to analgesia and motor facilitation. (**A**) Epidural or transcutaneous stimulation involves large-diameter somatosensory afferents located near the dorsal root entry zone (DREZ) and dorsal column pathways and therefore biases segmental networks and propriospinal networks that combine residual descending drive with afferent feedback. This mechanism is able to gate sensorimotor transmission, reduce recruitment thresholds in pools of motor neurons, and also enable the volitional production of motor output; simultaneously, a parallel dorsal horn pathway depicts the gating of nociceptive transmission. Circuit-based readouts include ECAP (which measures dorsal column recruitment), MEP and DTI (which measure the integrity of the descending pathways), H-reflex modulation (which measures segmental excitability), and the EMG coherence/muscle synergies (which measure motor output organisation). (**B**) The results are measured on very different temporal scales: there is rapid and reversible state modulation (seconds–minutes), and training-coupled adaptation (over days to weeks) via Hebbian-like and non-Hebbian processes. The human evidence is largely indirect, and functional gains tend to manifest during stimulation with differences in retention that occur upon discontinuing stimulation.

Spinal cord stimulation (SCS) impacts motor output by altering the sensory–motor interfaces that regulate segmental excitability [10]. Instead of operating solely through dorsal column stimulation, SCS seems to modify the effective gain of both proprioceptive and cutaneous projections of afferent input onto interneuronal pools, thus modulating the integration and relay of sensory information to motor neurons [11,12]. Though this awareness is based mainly on preclinical research, indirect evidence of a comparable capacity to re-modulate sensory–motor gating has been found in humans through changes in reflex modulation and recruitment patterns in the course of stimulation [13,14] (Figure 1).

The evidence is most consistent in incomplete spinal cord injury, where stimulation can unmask or amplify subthreshold descending commands and enable task-specific motor responses [15]. In contrast, anatomically complete or severe lesions show more limited recruitment of volitional motor output, underscoring the contribution of spared corticospinal and other descending pathways to SCS-enabled facilitation [16]. Accordingly, lesion morphology and residual network integrity should be considered when interpreting mechanistic and clinical effects.

Beyond afferent gain, SCS may also reconfigure interactions within excitatory and inhibitory interneuronal circuits that integrate sensorimotor signals [17]. Experimental studies describe changes in segmental reflex excitability, afferent–efferent coupling, and occasional central pattern generator-like activity; however, the functional relevance of pattern-generating mechanisms for human motor output remains uncertain [18,19]. Species differences in spinal circuit organisation further limit direct extrapolation [20]. Overall, the available evidence favours network-level reweighting and a permissive excitability state that increases responsiveness to descending commands and peripheral feedback, rather than the robust recruitment of a standalone locomotor generator. Mechanistic limitations and the indirect nature of human biomarkers are discussed in Section 2.6 [21,22].

### 2.2. Rationale for a Functional Restoration Framework

The repositioning of spinal cord stimulation (SCS) from nociceptive modulation toward functional restoration marks a key inflection in neuromodulation research [23], driven by evidence that the injured spinal cord and associated supraspinal pathways retain residual integrity and the capacity for activity-dependent reorganisation, even in chronic stages [24,25]. Preclinical studies across models suggest that epidural and non-invasive stimulation can increase interneuronal responsiveness and facilitate transmission through spared corticospinal and propriospinal pathways [26], with improvements in voluntary activation and postural control in experimental preparations [27]. Limited human observations point to similar principles, particularly in incomplete injuries when stimulation is paired with task-specific training [28].

Clinically, structured protocols combining SCS with high-intensity locomotor or upper-limb rehabilitation have enabled a subset of individuals with chronic spinal cord injury or post-stroke hemiparesis to regain voluntary motor behaviours previously considered unattainable [29], although reproducibility often depends on controlled settings, intensive therapist input, and the presence of spared descending pathways [30]. Simultaneously, the precision and stability of neuromodulatory delivery are being pushed beyond the limits of traditional systems using contemporary devices that combine high-frequency or burst stimulation paradigms with adaptive closed-loop control [31,32]. The motivation to recover motor autonomy is reflected in the underlying clinical rationale: it is universally considered that motor autonomy is a key determinant of quality of life following an injury to the spinal cord or stroke.

A conceptual framework of restoration further extends multimodal therapeutic approaches to include transcranial magnetic stimulation (TMS), transcranial direct-current stimulation (tDCS) in addition to peripheral nerve stimulation, and robotic-assisted training, which can synergistically interact with spinal cord stimulation (SCS) in simultaneously modulating cortical excitability and spinal sensorimotor integration [33]. In addition, there is growing evidence that is expanding restorative SCS to include cervical lesions and other neurological conditions besides thoracic injuries [34,35]. Irrespective of these developments, there remain salient challenges, such as the heterogeneous clinical outcomes, the relatively limited direct mechanistic evidence in human beings, and practical barriers to deployment [36], which necessitate cautious interpretation and the development of more clear criteria for patient selection and deployment.

### 2.3. Hebbian Plasticity and Activity-Dependent Strengthening

Hebbian plasticity, which refers to enhanced connections between synapses through a temporal association between the activity in the presynaptic input and the postsynaptic activity, provides a conceptual foundation for the likelihood of spinal cord stimulation (SCS) being able to strengthen motor circuitry, owing to the coincidence of the activity in the descending motor intention and the afferent feedback signals on shared populations of neurons [37,38]. This theoretical framework underpins activity-dependent protocols that use electrical stimulation and voluntary effort or training to target a task to coordinate exogenous neuronal input and endogenous motor drive [39].

Experimental evidence from rodent models shows that a precisely timed SCS can potentiate propriospinal and premotor interneuronal connections, which generate more dependable motor outputs as the training progresses and is repeated in sequence [40]. In human subjects, there is less evidence (although much indirect evidence), which includes the change in the reflex excitability, electromyographic coherence change, and task-dependent cortical and spinal activity patterns as a result of stimulation [41]. Most commonly, clinically, improvements are reported in patients who have some residual descending connectivity, whereas others plateau or even deteriorate with the implementation of intensive paired protocols [42]. Hebbian-like effects, where present, likely interact with broader state-dependent mechanisms (see Section 2.6) [43,44,45].

### 2.4. Non-Hebbian Plasticity, Homeostatic Regulation, and Neuromodulatory Processes

Beyond spike-timing-dependent Hebbian plasticity, SCS may drive adaptive change through non-Hebbian mechanisms that regulate circuit stability and excitability at the cellular and network levels [46]. Homeostatic plasticity (including synaptic scaling, metaplasticity, and shifts in intrinsic neuronal excitability) can adjust global network gain during sustained neuromodulatory input [47], although its operational relevance in humans is supported mainly by indirect, longitudinally sparse physiology [48].

Preclinical studies suggest that prolonged or repetitive stimulation can bidirectionally scale synaptic efficacy, alter ion-channel expression, and shift action-potential thresholds, changes that may increase the probability of recruiting interneuronal circuits relevant to motor coordination [49]. Compared with Hebbian processes, these mechanisms act over longer timescales and are less dependent on millisecond-level pairing, consistent with a state-setting role rather than synapse-specific reinforcement.

SCS may also interact with neuromodulatory and neuroimmune milieus that shape plasticity. Experimental studies involve glial activity changes, cytokine signalling, and the neuroinflammatory state [50], and hypotheses on pathways include BMP4–Smad signalling in animal models though not in humans [51]. In parallel, adjustments of serotonergic and noradrenergic tone—being notable in spinal cord injury models—could modulate membrane excitability and transmission across residual sensorimotor circuits during the neuromodulatory stimulation [52].

Non-Hebbian mechanisms at the network level have been linked to changes in the excitatory–inhibitory equilibrium, stronger interneuronal synchrony, and altered oscillatory activity [53], although such changes have traditionally been estimated in humans by surface EMG coherence or kinematic proxy, hence limiting mechanistic resolution [54]. Section 2.6 addresses methodological and translational limitations, such as species and protocol variability, and the use of surrogate biomarkers [55,56].

### 2.5. Excitability Priming and State-Dependent Modulation

In addition to synaptic plasticity, spinal cord stimulation (SCS) can lead to motor output through the temporary, rapid, and reversible modulation of spinal excitability in the form of excitability priming [57]. This priming has been hypothesised to mirror the temporary changes in membrane potential, interneuronal gain, or recruitment thresholds that increase responsiveness to afferent input and residual descending commands without requiring the long-lasting synaptic remodelling process [58]. One of the most notable observations in human beings would be that voluntary movement usually occurs during stimulation and fades away after stimulation has ceased, which is also typical of state-dependent facilitation.

Priming should not be framed as being a process of reactivating dormant circuits; the effect of SCS, assumed to be on the signal-to-noise properties of already established pathways, is to increase the chances that weak residual conduction will be able to control motor pools. The basis of indirect evidence is the changes in stimulation-related EMG coherence, muscle recruitment, and reflex responsiveness [59,60,61]. The clinical persistence of such effects and their independence of concomitant learning or long-term plasticity are still unknown and are discussed, along with more general mechanistic constraints, in Section 2.6.

### 2.6. Mechanistic Uncertainties, Evidence Limitations, and Translational Constraints

Although there is a growing academic curiosity over the use of spinal cord stimulation (SCS) as a tool to regain motor ability, the mechanistic basis of SCS in humans is largely conjectural. Most information in detailed physiological understanding has been brought forth by preclinical models, whilst human experiments strongly rely on surrogate readouts—electromyographic coherence, reflex excitability, and stimulation-enabled locomotor patterns—that are insufficient to directly resolve causality on a synaptic or circuit basis. The small cohorts of the study, diverse injury profiles, and high variability of stimulation programming are additional limitations to improving the reproducibility and generalizability of the mechanistic conclusions between studies.

Translational impediments include species-specific differences. Robust locomotor pattern-generating activity and clearly defined interneuronal organisation are common in rodent models; the human corticospinal control is much more complex, and the generation of intrinsic motor patterns is less easily observed, making it unclear how to map a specific mechanism [55]. Plausible hypotheses based on animal models, including the BMP4–Smad cascade, involve molecular or glial pathways, which have not been substantively proven in humans.

The second methodological problem is attributing the observed effects. Hebbian reinforcement, non-Hebbian homeostatic readjustments, and state-dependent shifts in excitability can occur in SCS to produce the same behavioural signal. These majorly impact the clinical effects because the gains in performance that can be attained with stimulation may not always be replicated into stimulation-independent recovery. Many reports describe gains that decrease with the withdrawal of stimulation, and durable improvements after training are inconsistently repeated; they are often based on highly structured procedures [62]. The heterogeneity of responses is further related to the level and chronicity of the lesion, the remaining descending connectivity, the spasticity, the position of the electrode, and the dosage and timing of ongoing rehabilitation.

The next phase of advancement will lie not so much in attributing more mechanistic labels but in using more research designs which separate temporary or state effects and more long-term physiological adaptations, using harmonised outcome measures and stratifying the patients based on the integrity of the remainder pathways.

## 3. Technological Foundations and Engineering Architectures of Motor-Restorative Spinal Cord Stimulation

### 3.1. Evolution of Stimulation Paradigms: From Analgesic Waveforms to Circuit-Targeted Modulation

Spinal cord stimulation (SCS) has moved from classic tonic, paraesthesia-based waveforms toward a broader set of paradigms informed by spinal physiology and device programmability. Tonic stimulation, rooted in gate-control principles, was engineered to recruit large-diameter dorsal column afferents and was developed almost entirely around analgesic endpoints [63]. Later innovations—burst stimulation, 10 kHz high-frequency stimulation, and differential target multiplexing—expanded pain options by altering perceptual experience and, potentially, engaging alternative spinal pathways [64,65]. These approaches, however, were not originally designed to facilitate motor recovery or circuit reorganisation.

Waveforms likely interact with spinal networks in distinct ways, although the mechanisms remain incompletely resolved. Tonic stimulation produces a relatively predictable pattern of dorsal column recruitment, whereas burst paradigms introduce temporally clustered input that may reshape interneuronal processing beyond simple afferent gating. High-frequency stimulation suppresses paraesthesia and may act through different field interactions within the dorsal horn, while differential multiplexing has been proposed to influence glial–neuronal signalling, supported mainly by early-stage data.

Pain-optimised waveforms cannot be assumed to translate to motor rehabilitation. Motor-restorative protocols developed in landmark programmes differ from FDA-approved pain therapies in amplitude, spatial targeting, timing strategies, and their coupling to task-specific training [66]. The evidence base also diverges: analgesic indications are supported by large randomised trials, whereas motor-oriented stimulation has largely been evaluated in small, highly selected cohorts using centre-specific montages [67,68]. For motor applications, the key practical gap is not the absence of waveforms but the lack of a shared reporting and optimisation framework to compare parameter choices across studies.

### 3.2. Lead Architecture, Anatomical Targeting, and Field Shaping in Motor-Restorative SCS

Lead design and positioning are critical factor in substrate recruitment during spinal cord stimulation (SCS). Paddle leads give rise to a more directional and spatially confined electric field, compared to percutaneous cylindrical leads; percutaneous leads are easier to implant but are more likely to give a wider current spread with lower selectivity. This control is further refined by high-density segmented arrays, which permit current steering across contacts as well as rostrocaudal or lateral axes in order to allow finer targeting of distributed pathways [69,70].

The requirements are different based on clinical activities, i.e., pain or motor restoration. In pain-based SCS, pain-related therapeutic strategies are typically targeted at midline dorsal columns; motor-restorative strategies focus on rostrolateral localization next to dorsal-root entry zones or specific afferent fascicles in order to bias sensorimotor circuits. Limited experimental and clinical studies show that overlapping electric fields over specific spinal segments have the potential to better interact with interneuronal networks that may be associated with locomotor or upper-limb regulation [71,72], though the optimal human targets—and their dependence on spared descending projections—remain incompletely mapped.

Field shaping is further constrained by posture-dependent impedance changes, which can shift effective recruitment during sitting, standing, and movement [73]. Inter-individual anatomy, cerebrospinal fluid thickness, and lead migration add additional variability, complicating replication across centres and studies. Yet many reports provide limited detail on contact geometry, impedance, or field modelling, reducing interpretability and comparability. Finally, alternative targets such as the dorsal root ganglion offer highly focal afferent modulation and strong pain efficacy, but their role in volitional motor recovery is still unclear [74,75], and hardware constraints (contact density, fixation stability, battery capacity) limit the achievable precision. Overall, lead architecture and anatomical context are not technical footnotes; they are central variables that should be reported and tested explicitly in motor-restorative SCS.

### 3.3. Closed-Loop, Sensor-Guided, and ECAP-Based Neuromodulation Systems

Closed-loop SCS adjusts stimulation in real time using physiological or behavioural feedback, an attractive concept for motor restoration where optimal settings vary with posture and movement. Current approaches cluster into ECAP-guided control, peripheral sensor-guided stimulation, and data-driven (machine learning) adaptation.

ECAP-based control is the most mature and is already used commercially for pain therapy [76]. By tracking dorsal column fibre recruitment, ECAPs can stabilise the effective dose across impedance and posture changes. For motor-restorative paradigms, however, ECAPs remain an imperfect proxy because they largely index large-diameter afferent activation, are vulnerable to motion-related artefacts and lead-position variability, and have not yet been shown in controlled studies to improve motor outcomes.

Sensor-guided systems bypass some of these limits by using kinematics, ground-reaction forces, or surface EMG to align stimulation with gait phase or voluntary intent. Phase-synchronised stimulation, according to early laboratory studies, has the capacity to regulate locomotor timing and support task performance when residual voluntary effort is present [77,78]. The real-life limitations of such interventions are quite pronounced because they require external hardware, have a calibration burden, and are sensitive to noise, fatigue, and sensor motion. As a result, the existing empirical evidence is mostly based on small and well-controlled cohort studies.

Theoretically, machine learning controllers would support the navigation of high-dimensional parameter spaces and would reduce the programming requirements of clinicians, but these remain largely preclinical. The main challenges are the limited training datasets, inter-device and anatomical heterogeneity, low interpretability and failure-mode management, and unclear regulatory expectations over autonomous or semi-autonomous control.

In the short term, the field needs a reduction in unreliable general assertions and a focus on testable linkages: that is, what feedback indicators are consistently accurate predictors of a clinically significant motor outcome and what the most effective rehabilitation dosage is. It will require the standardisation of reporting and trials, and the ability to separate “state-contingent performance” from durable gains that are independent of stimulation.

### 3.4. Multimodal Integration: Combining SCS with Cortical, Peripheral, and Robotic Interventions

Multimodal rehabilitation pairs spinal cord stimulation (SCS) with interventions that act upstream or downstream of the spinal cord, aiming to couple segmental excitability with a stronger descending drive and richer sensory feedback. Alongside SCS, TMS/tDCS, peripheral stimulation (PNS/FES/EMG-triggered protocols), and robotic gait systems target different nodes of the motor hierarchy and are most feasible when their timing is aligned with voluntary effort or a task phase [79].

Cortical–spinal pairing (SCS with TMS or tDCS) is used to raise the corticospinal drive while the spinal cord is in a more excitable state, potentially improving transmission through partially preserved pathways. Small, single-centre studies report task-contingent gains during combined stimulation, consistent with timing-sensitive facilitation across levels of the neuraxis [8,80]. The limitation is not the rationale but the protocol spread: studies vary in timing windows, intensity, and concurrent training, and often use non-comparable outcomes. As a result, no reproducible “standard” cortical–spinal pairing rule has yet emerged for motor rehabilitation.

Peripheral approaches aim to sharpen the link between intent, afferent feedback, and muscle activation. PNS, FES, and EMG-triggered stimulation can enrich proprioceptive inflow and enforce task-specific recruitment; when integrated with SCS, they may better couple residual descending commands to movement. Current evidence is still mostly based on feasibility studies and case series, and shows great diversity in parameter selection and electrode location as well as rehabilitation dosage [81].

A major barrier outside specialised research settings when multiple devices are involved is the ability to perform session-to-session recalibration and the synchronisation of various devices. Gait training assisted by robots can offer other contributions, standardising kinematics, increasing the number of repetitions, and decreasing the use of compensatory mechanisms, while SCS modulates spinal excitability. Early studies suggest that the simultaneous use of robotics and SCS can improve step consistency and involvement in the performed activity; although most of the data are obtained in laboratory conditions, the effect on practical functional autonomy remains to be determined. The financial expense, infrastructural needs, and staffing needs have remained the major impediments to scale-up.

Together, multimodal interventions are most convincing, as they specify a verifiable rule of coupling (i.e., what signal is synchronised to what phase, with what dosage of training), and when the result measures include stimulation-off retention as well as stimulation-on performance. The need to establish whether multimodal neuromodulation has a long-lasting benefit over well-delivered SCS plus rehabilitation will require multicentre studies with the use of harmonised reporting as well as common endpoints.

### 3.5. Practical Engineering Constraints and Real-World Implementation Challenges

System reliability limits the clinical outcome of motor-restorative spinal cord stimulation (SCS) just as much as biological plausibility. Field geometry may vary with time due to lead migration (especially with percutaneous systems), posture- and anatomy-dependent variations in the cerebrospinal fluid thickness, and scarring, which can all diminish the spatial selectivity on which motor protocols rely [82]. Power constraints during multi-channel high-duty-cycle operation also limit long-term performance, alongside risks of infection, component fatigue, and susceptibility to electromagnetic interference—failure modes that are less forgiving when the stimulation is required to be precise and not just perceptible.

Motor-oriented programming is also not scalable in its current form. Spatiotemporal recruitment can frequently be effective through iterative contact and waveform parameter tuning, and the same settings can result in differences between sessions because of variability in impedance, fatigue, and afferent excitability with posture and training context. This programming overhead, together with the lack of reporting of montages and device–tissue conditions, results in a very strong centre effect, in which repeatable benefits are concentrated in a limited set of specialised groups [83]. What remains practical is thus reliant on two deliverables, which are adaptive programming that stabilises the dose under real-world variance, and a minimal reporting standard making configurations across centres comparable.

The prerequisites of scalability are also patient- and pathway-level in nature. The level and chronicity of the lesion, and extent of corticospinal/reticulospinal sparing all influence outcomes, while severe spasticity, disuse atrophy, and a lack of endurance can prevent the acquisition of stimulation-enabled synergies even in the case of technically sufficient stimulation. Most of the published protocols expect access to intensive therapist supervision and enabling infrastructure (robotics, high-fidelity tracking), which is currently not routinely available beyond tertiary environments; without a deliverable rehabilitation dose capable of being delivered by general services, population-level translation will remain limited.

Lastly, the cost, regulations, and safety affect implementation. The accumulated cost of implantation, monitoring, reprogramming, and high-intensity rehab is an institutional impediment wherein reimbursement is unclear. Adaptive and AI-governed control brings in regulatory examination into validation, responsibility, and protection against drift or unsafe autonomous actuation. To achieve a viable pathway to scale, there is a need for engineering refinement, a pragmatic service model, and governance that will assure safety without aggregating access in a small group of well-endowed centres [84].

## 4. Specific Disease Contexts and Functional Outcomes

Section 4 provides a structured overview of the clinical applications of spinal cord stimulation (SCS), organized according to the maturity and strength of available evidence. Chronic neuropathic pain represents an established indication supported by randomised controlled trials and long-term real-world data. In contrast, motor-restorative applications—particularly in spinal cord injury and stroke—remain investigational, with evidence derived primarily from early-phase clinical studies and specialised translational programmes. Emerging applications in other neurological disorders are exploratory. This evidence-based stratification is intended to support clinically grounded interpretation and facilitate responsible translation into practice.

### 4.1. Spinal Cord Stimulation for Pain Syndromes: Modalities, Evidence, and Clinical Nuance

Spinal cord stimulation (SCS) has the strongest evidence base in the context of chronic neuropathic pain, including failed back surgery syndrome (FBSS), complex regional pain syndrome (CRPS), and refractory radiculopathy. Among all current applications of SCS, these pain indications are considered established, supported by multiple randomised controlled trials and long-term observational studies. In these indications, randomised trials support clinically significant short- to intermediate-term decreases in the severity of pain along with improvements in quality of life [85]. However, clinical outcomes vary according to pain phenotype and patient characteristics, and real-world effectiveness depends strongly on appropriate waveform selection and careful patient selection.

The nature of waveforms differs in tolerability and, in selected settings, efficacy. Traditional tonic stimulation makes use of dorsal column Aβ fibres resulting in paraesthesia; it is the most used modality in FBSS but it has certain limitations of discomfort or habituation. Burst paradigms produce trains with temporality and often achieve analgesia without conscious paraesthesia, yet these outcomes are inconsistent across populations. High-frequency 10 kHz stimulation has demonstrated superiority over conventional tonic stimulation in landmark randomised trials, offering paraesthesia-free analgesia in selected patient groups, although its precise mechanisms remain incompletely understood [86,87]. Differential target multiplexing has been proposed to work through the interactions between glia and neurons, but the evidence is currently at a preliminary level. Importantly, no single waveform has proven universally superior, and cross-trial comparisons are constrained by differences in study design, follow-up duration, and inclusion criteria.

CRPS emphasises the non-interchangeability of pain indications. Its mixture of disrupted somatosensory integration, autonomic dysfunction, and common comorbidity with psychiatric disorders contributes to heterogeneous responses to treatment; the maintenance of benefit is less predictable than in FBSS even with an ideal location of the leads and programming. Poor outcomes are linked to longer disease duration, severe central sensitisation, and serious emotional distress [88]. These factors underscore the importance of rigorous patient selection and realistic preoperative counselling (Table 1).

Treatment durability is also influenced by patient-related factors. Sustained analgesia is more likely in patients with predominantly neuropathic pain, shorter symptom duration, and limited psychological comorbidity, whereas high opioid dependence and mixed nociceptive–neuropathic phenotypes often predict weak or less enduring gain [23]. The efficacy in some patients wanes during long-term follow-up, or there is a need to perform explantation, highlighting the clinical importance of mechanism-based phenotyping and expectation management.

### 4.2. Motor Restoration After Spinal Cord Injury: Evidence, Patient Profiles, and Clinical Realities

The therapeutic use of spinal cord stimulation (SCS) in motor restoration among those with spinal cord injury (SCI) has remained largely experimental. The majority of published “breakthroughs” come from a reduced number of specialised facilities that apply extensive, multimodal rehabilitation programmes on carefully selected individuals, usually with incomplete injury and evidence of residual descending connectivity. Both transcutaneous and epidural stimulation can enable stepping-like activity, improve postural control, and allow voluntary activation under supervised conditions, but these results are often conditional on continued stimulation and rarely produce long-term and stimulation-independent motor autonomy.

The current body of evidence is limited by the sample size as well as the challenges of attributing the effects observed to particular components of the intervention. Stimulation is often combined with high-dose therapist input, body-weight support, or robot assistance in epidural programmes, making it difficult to separate the effect of stimulation per se when used in training. Transcutaneous methods are less invasive but have inconsistent efficacy and frequently small, inconsistently repeated effects. Across both modalities, studies vary in electrode arrangement, waveform, timing strategy, and dose in rehabilitation, thus making comparisons between the studies difficult and preventing a comparable programming strategy across modalities [7]. Most of the outcome measures are acquired in laboratory settings, and little evidence shows that the benefits persist in an unsupervised community setting.

The characteristics of patients are very critical in clinical responsiveness. The strongest gains are found in those subjects with AIS class C/D injuries, where identified spared corticospinal or propriospinal pathways can be activated, whereas AIS class A/B injuries generally have few volitional gains, even when stimulation triggers reflexive or patterned activity. Other factors that also influence the clinical course include the chronicity of the injury, spasticity, denervation and muscle reserve, autonomic instability, and the ability of the patient to undertake strenuous and repeated training sessions. Translation is therefore constrained not only by a lack of large, randomised trials and the limited extent of long-term follow-up but also by the infrastructure and staffing requirements of many protocols. As such, the practical next step is explicit stratification criterion testing and the evaluation of outcomes that matter for scale—stimulation-off retention and real-world functional performance—and not just stimulation-on performance.

### 4.3. Post-Stroke Motor Enhancement with Spinal Cord Stimulation: Early Evidence and Translational Barriers

Recent proof-of-concept studies establish the basis of the use of spinal cord stimulation (SCS) in the post-stroke rehabilitation process, especially in upper-limb rehabilitation. Such works prove that SCS has the capacity to amplify task involvement and enable stronger voluntary motions among selected participants. Even so, the most commonly mentioned evidence, such as a 2022 study in Nature Medicine, relies on tiny uncontrolled cohorts, uses highly individualised electrode placement, and relies on specialised laboratory facilities, thus providing a signal rather than a scalable protocol [89].

Available evidence shows that motor gains observed in various studies are mostly dependent on the availability of continuous stimulation. Wrist extensions, grip strengths, and reach-to-grasp control become best evidenced in active SCS, and decline quickly with withdrawal, which suggests a temporary enhancement of spinal responsiveness to descending cortical commands in the motor circuitry instead of a lasting reorganisation of the motor circuitry. Heterogeneity in the target sites of stimulation, stimulation parameters, dosage, and timing of task practice further limits the interpretative scope in that the effects of a stimulation intervention cannot be isolated within the larger training context.

Responsiveness now seems to be a factor for patient-specific residual corticospinal integrity. People who show spared tract architecture or clearly sustained voluntary activation are likely to achieve higher functional improvements, but extreme tract disruption, prominent severe spasticity, and maladaptive synergies are associated with poor results. The inherent divergence of stroke pathology, which includes the lesion topography, sensory loss, and cognitive losses, including visuospatial neglect, and systemic comorbidity adds to the difficulty of transferring a neuromodulatory boost to functional competence in the long term. Emerging areas of translational shortage are noticeable: there is a lack of randomised controlled trials; the sustainability of improvements beyond stimulation-on performance have not been sufficiently described; and the safety of cervical epidural SCS in stroke comorbid cohorts has not been systematically defined. Progress requires multicentre studies requiring explicit selection, standardised outcome measures, and a design that can distinguish between state-dependent facilitation and true retention after stimulation is removed [90].

### 4.4. Patient Selection and Predictors of Response Across Pain and Motor-Restorative Indications

The efficacy and durability of spinal cord stimulation (SCS) depend less on the device itself than on the biological substrate and the patient’s capacity to translate stimulation-enabled performance into trained function. Across indications, selection is best framed around three domains: (i) preservation of the target pathway to be modulated, (ii) symptom phenotype (what problem is being treated), and (iii) behavioural and rehabilitative feasibility.

In chronic pain, the most reliable responders are those with predominantly neuropathic, anatomically localised symptoms—classically failed back surgery syndrome and radicular neuropathic pain—where the pain generator and distribution are stable and consistent. Mixed nociceptive/biomechanical pain phenotypes predict weaker benefit, and psychological/behavioural factors such as high catastrophising, untreated affective disorders, and significant opioid dependence further reduce durability [91]. CRPS is more variable because central sensitisation and autonomic features often coexist with psychosocial burden; a shorter disease duration and lower psychological distress tend to be more favourable, whereas longstanding disease and pronounced affective distress often attenuate long-term analgesia.

For motor restoration after spinal cord injury, preserved descending connectivity is the dominant prerequisite. AIS C/D injuries show the most consistent stimulation-enabled gains because residual corticospinal/propriospinal pathways can be recruited, whereas AIS A/B injuries rarely achieve meaningful volitional recovery despite stimulation. Chronicity, spasticity burden, autonomic instability, and muscle reserve then shape whether a patient can tolerate stimulation and complete the training dose needed to consolidate performance.

In post-stroke cohorts, the response to cervical SCS similarly tracks residual corticospinal tract integrity and voluntary drive: the presence of motor-evoked potentials or imaging evidence of partially spared projections is more favourable, while severe abnormal synergies, major sensory loss, and higher-order deficits (e.g., neglect, apraxia) often prevent the translation of stimulation into functional skills. Participation constraints—pain, contractures, and limited cognitive/physical endurance—are practical gatekeepers because most protocols require repeated task-specific practice [66].

Across indications, favourable profiles converge on preserved pathway integrity, shorter chronicity, lower psychological burden, and sufficient cognitive/physical capacity to engage in structured rehabilitation; however, formal stratification frameworks remain underdeveloped, particularly for motor-restorative use. Progress now depends on standardised selection criteria, harmonised outcomes, and prospective multicentre studies incorporating imaging or electrophysiological biomarkers; until then, careful individual assessment and explicit expectation setting remain essential for ethical implementation.

### 4.5. Outcome Measures, Durability of Benefit, and Real-World Effectiveness

The measurement of spinal cord stimulation (SCS) results is designed to reflect the therapeutic goals, but the current measures of the conditions offer an incomplete analysis of clinical effectiveness. With regard to chronic pain, the efficacy measures tend to be visual analogue scales (VASs) or numeric rating scales (NRSs), accompanied by disability indices, including the Oswestry Disability Index (ODI), quality-of-life measures, and global impression scales, one of which is the Patient Global Impression of Change (PGIC) [92]. Despite their sensitivity to short-term analgesic actions, the instruments are influenced by the psychological condition, context, and expectancy effects and do not always distinguish between pain intensity reduction and functional capacity improvement.

There are different challenges with motor-restorative interventions. Ameliorations often turn out to be stimulation-contingent performance instead of independent action, which requires measuring the kinematics, electromyography, and activity-specific measurements of voluntary movements. The cross-trial synthesis is hampered by the heterogeneity of endpoints and reporting conventions across studies, in addition to the fact that the absence of harmonised outcome frameworks limits the interpretability of findings across different stimulation paradigms and rehabilitation intensities.

Analgesic and motor indications are also different with regard to durability. The clinically meaningful advantages are well documented at 6–12 months after the implantation procedure in the pain populations; nevertheless, in the long-term follow-up, attrition, the development of tolerance, or device explantation is detected in a subgroup of the population. Follow-up tends to be brief in motor cohorts, and gains tend to fade when stimulation is withdrawn, suggesting that much of what is reported may be a state-dependent facilitation and not long-term neuroplastic adaptation. Few studies prospectively measure stimulation-off retention, leading to poorly defined benefit sustainability.

There is another gap between the efficacy of laboratory work and real-world effectiveness. Although analgesic SCS is observed to be relatively stable outside controlled trials, the results are dependent on programming experience, adherence to follow-up, and psychosocial comorbidity. To restore the motor, the majority of recorded increases occur in well-controlled settings, such as robotic interfaces, real-time motion capture, and intensive therapist input, which are not comparable to community systems of rehabilitation [93]. Therefore, the application of these effects to daily life activities like ambulating independently or taking care of oneself is not consistently generalised, particularly in the presence of fatigue, environmental variability, and cognitive load.

Advancements on this front require the use of indication-specific assessment models. In the case of pain, composite endpoints that combine the intensity of pain, functional status, the use of opioids, and quality of life would be more appropriate measures of therapeutic worth than single-dimensional measures. In the case of motor indicators, the neurophysiological indicators of engagement in pathways should be combined with task-based performance and participation indicators that are aligned with the International Classification of Functioning, Disability and Health (ICF). Notably, future experiments should predetermine and provide stimulation-on and stimulation-off results to distinguish between short-term facilitation and long-term adaptation. The multicentre harmonisation of reporting standards—including the stimulation parameters, dosages of rehabilitation, and follow-up durations—will be necessary in order to determine the reproducibility of the observed effects outside of specialised centres.

### 4.6. Structured Summary of Major Clinical Case Series and Translational Status

To provide a clearer view of the current clinical landscape, Table 2 summarises representative randomised trials and major case series across pain and motor-restorative indications. The table highlights differences in study design, cohort size, the durability of reported effects, and overall evidence maturity.

For chronic neuropathic pain, including failed back surgery syndrome, complex regional pain syndrome, and painful diabetic neuropathy, spinal cord stimulation is supported by multiple randomised controlled trials and multicentre studies. In these populations, responder rates of approximately 50–70% for clinically meaningful pain reduction have been reported, with sustained benefit in many patients at 6–12 months, although long-term attrition and device explantation occur in a subset. In this context, SCS can reasonably be regarded as an established therapy within defined patient-selection criteria.

By contrast, motor-restorative applications remain at an earlier stage of development. In spinal cord injury, epidural stimulation programmes conducted in specialised centres have demonstrated the re-emergence of voluntary stepping, improved trunk stability, and stimulation-enabled standing in carefully selected individuals, particularly those with incomplete injuries and preserved descending connectivity. However, most cohorts remain small, typically involving fewer than 10 participants per program, and gains are often dependent on ongoing stimulation and intensive rehabilitation. Consistent demonstration of stimulation-independent retention remains limited.

Transcutaneous spinal stimulation for motor recovery has been evaluated in small pilot cohorts and feasibility studies. Although improvements in EMG recruitment patterns and stepping-like activity have been described, reproducibility across centres and durability after the withdrawal of stimulation are variable. These findings support biological plausibility but do not yet constitute standardised clinical protocols.

Early investigations in post-stroke hemiparesis, including cervical epidural stimulation combined with task-specific training, have shown stimulation-contingent improvements in grip strength and upper-limb control in small uncontrolled cohorts. At present, these data represent proof-of-concept observations rather than established treatment pathways. Evidence in Parkinson’s disease and multiple sclerosis remains exploratory and based on small case series.

Taken together, the current evidence base is uneven across indications. Chronic neuropathic pain represents the only domain supported by randomised trials and longer-term outcome data. In contrast, motor-restorative applications in spinal cord injury and stroke are best characterised as investigational, with encouraging but protocol-dependent results. Clear differentiation between stimulation-enabled performance and durable recovery will remain essential as the field advances toward larger multicentre trials and broader clinical translation.

## 5. Future Directions in Neuromodulation: Scientific Priorities, Technological Challenges, and Pathways to Clinical Translation

### 5.1. Mechanistic Priorities: Clarifying Circuit-Level Pathways and Biomarkers

Although spinal cord stimulation (SCS) has been clinically feasible, the circuit-level mechanism behind the treatment has not been fully elucidated. Specifically, the comparative importance of transient excitability changes for long-term neuroplastic changes has not been clearly delineated, although the distinction directly relates to expectations about the maintenance of treatment and protocol design. Therefore, the parameter selection in recent practice is mostly empirical because no single model is yet available that connects particular waveforms, amplitudes, and spatial configurations with particular spinal or corticospinal circuit targets.

There are three mechanistic axes, which need to be elaborated. First, the preferred populations of interneurons recruited during motor-facilitating stimulation are unknown, and it remains unclear whether the net effects depend on the balance between excitation and inhibition in segmental networks. Second, the time-dependent convergence between the descending corticospinal drive and the stimulus-regulated dorsal root afferent signal has been inadequately defined, especially in task-dependent and dynamic conditions. Third, there are no longitudinal experiments to determine whether repeated stimulation causes the reorganisation of spinal or supraspinal circuits, or whether the numerous gains actually observed are simply reversible modulation of states imposed on remaining pathways. Answering these questions will require joint human and preclinical research consisting of electrophysiology, computational modelling, and imaging in designs that are explicitly capable of separating the effects of state and long-term adaptation.

Progress toward precision neuromodulation is also based on the validated biomarkers. Currently, there is no consensus regarding a framework that has been put in place to rank patients based on pathway integrity or the chance of responding. Measures of corticospinal tract structure, motor-evoked potentials, spinal-evoked compound action potentials, reflex modulation profiles, and surface electromyography-derived muscle synergy patterns are candidate markers [94]. However, none of these metrics have been prospectively established to predict lasting clinical benefit and comparability is restricted by methodological heterogeneity between centres. Further investigations must thus investigate whether multimodal biomarker panels can differentiate between stimulation-enabled performance and long-term functional recovery and whether there is any ability of multimodal biomarkers to inform individualised targeting and parameter selection across a wide range of patient groups.

### 5.2. Technological Priorities: Refining Precision Stimulation and Adaptive Control

The future development of spinal cord stimulation (SCS) will be less about adding features than about addressing three overarching technological constraints: a lack of spatial selectivity, sensitivity to posture-related impedance variations, and an inadequate ability to modify parameters state-dependently. The existing systems are not yet able to provide the same consistency in circuit recruitment as the neural and biomechanical conditions vary, although they can provide more complex waveforms. A rational solution here is to use adaptive control, although safety thresholds and failure contingencies must be clearly defined before it is applied widely in clinical practice.

ECAP-guided systems are the most developed adaptive systems, mostly in pain indications. Through observing the dorsal column recruitment, ECAP feedback can be used to stabilise the delivered dose in response to posture and impedance change. Nevertheless, ECAPs provide only indirect information about the dynamics of interneurons of motor interest and are not able to clarify the interaction of stimulation with the descending drive. Advanced systems that involve sensor-guided methods such as the use of inertial units, surface EMG, or force platforms are more directly aligned with motor rehabilitation because they can enable phase-specific or task-contingent stimulation [95]. The practical constraints of the latter are the signal noise, calibration workload, effects of user fatigue, and dependence on the facilities that are often absent beyond specialised centres. Machine-learning strategies can offer a possible solution to the issue of parameter optimisation or intent decoding in the future but are too limited in terms of the training data used, risk of over-fitting, opaque decision-making, and lack of regulatory frameworks to be allowed into standard clinical practice.

Technical refinement is hence to be given priority. Hardware stability (lead fixation, contact density, charge efficiency, and the mitigation of posture-related field drift) needs to be achieved before more complicated control algorithms can be sensibly analysed. Computational modelling and multimodal physiological integration ought to be assessed versus reproducible functional endpoints as opposed to surrogate engineering metrics. In the case of adaptive systems, scalability requires explicit safety envelopes, human-in-the-loop control, and predefined responses to signal loss or model uncertainty. The level of technological sophistication will not guarantee clinical impact; what is needed is the thoughtful integration of engineering precision with validated biomarkers, reproducible results, and the constraints of routine care delivery.

### 5.3. Integrative Neurorehabilitation Ecosystems: Multimodal Synergy and Scalability

When the components are each involved in approaching a different bottleneck of the motor system and these components are promptly coordinated to respond to the behavioural event, multimodal neurorehabilitation is most defensible. Cortical stimulation therapies, including transcranial magnetic stimulation (TMS) and transcranial direct-current stimulation (tDCS), are capable of increasing corticospinal drive; spinal cord stimulation (SCS) can set segmental excitability and sensorimotor gating; and peripheral stimulation therapies, such as functional electrical stimulation (FES) and robotic systems, are capable of providing high-dose, high-feedback practices. Based on this, synergy should be described in terms of a coupling rule that determines which physiological signal to synchronise with an individual movement or volitional intention, as opposed to simply adding more devices.

**Figure 2:**
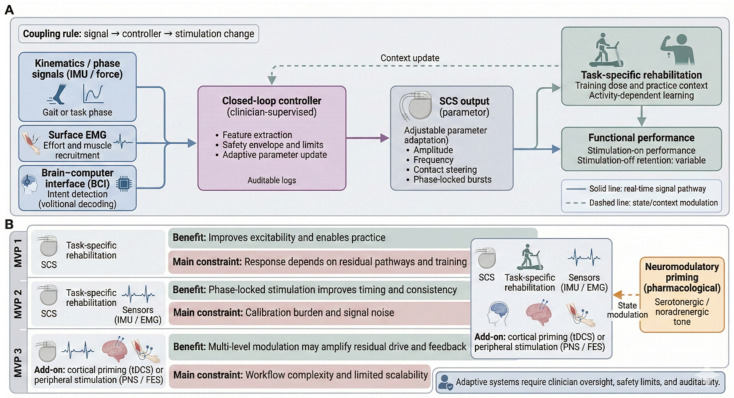
A coupling-rule framework for multimodal, closed-loop spinal cord stimulation and scalable implementation tiers. (**A**) Multimodal synergy has been conceptualised as an explicit rule of coupling: physiological or intent signals (kinematics/phase based on inertial measurement units or force sensors, surface electromyography, or brain–computer interface decoding) are integrated into a clinician-supervised closed-loop controller that can generate auditable logs. Task-specific rehabilitation provides the dose of the training and practice conditions, which determine functional performance, while the controller adjusts the spinal cord stimulation (SCS) output parameters, which are amplitude, frequency, contact steering, and phase-locked bursts, to match the stimulation to the task context. The results are divided into stimulation-on performance with variable stimulation-off retention. The solid lines represent real-time signal paths; the dashed lines represent the context/state input. (**B**) Minimum viable multimodal packages (MVP 1–3) demonstrate increasing degrees of integration for clinical scale-up: MVP1 contains SCS with task-specific rehabilitation, MVP2 contains added sensors (phase-locked control), and MVP3 contains optional add-ons (such as cortical priming (tDCS) or peripheral stimulation (PNS/FES)), with pharmacological neuromodulatory priming as a state modulator. Each level is a summary of the benefit expected and the major limitation to implementation. The different arrow colors are used to visually distinguish pathways among input sensing, controller output, stimulation delivery, and task/performance feedback; they do not denote separate experimental groups or statistical categories.

Such integration only has early empirical evidence, with most findings being from small and single-centre studies undertaken under controlled conditions. The reported benefits tend to be task-contingent and most predictable when stimulation is synchronised to particular movement stages or with regard to the extent of effort; nevertheless, the benefits cannot be interpreted because of the variability in electrode placement, pulse characteristics, timing plans, and rehabilitation dose. Due to the frequent lack of protocols and outcome measure harmonisation, it is difficult to compare across studies, and one cannot be sure which pairing rules are applicable outside specialised laboratories (Table 3, Figure 2).

The major impediment is scalability. Integrative protocols have some common requirements—multiple devices, recurring calibration, and concurrent clinician, engineer, and therapist input (which many rehabilitation services cannot afford in terms of workflow). Such resource demands have been found to focus resource access on a small number of centres and increase cost-based inequity. On the side of the patient, long sessions, exercise loads, and frequent adjustments reduce compliance and limit their relevance to highly motivated individuals. An empirically convincing near-term goal, then, is to specify a minimum viable multimodal package to maintain the coupling rule yet cut down the number of devices, configuration time, and staffing burden.

**Figure 3:**
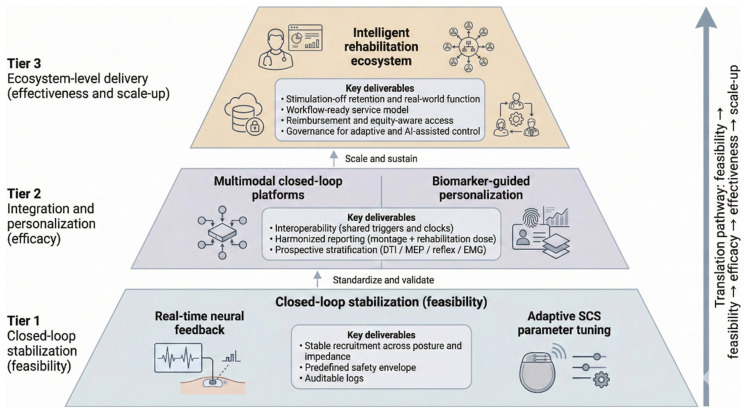
A three-tier translation pathway for next-generation closed-loop spinal cord stimulation in rehabilitation. The framework will be structured into a stepwise progression beginning with Tier 1, which is referred to as the feasibility, then the next one is referred to as the efficacy, and the last stage is referred to as the effectiveness and scale-up. Tier 1 highlights real-time neural feedback and adaptive spinal cord stimulation (SCS) parameter optimisation, whose major deliverables will include stable recruitment across posture/impedance, pre-specified safety envelopes, and auditable logs. Tier 2 is based on multimodal closed-loop platforms and biomarker-enabled personalisation, where the consideration of interoperability, harmonised stimulation montage, and rehabilitation dose reporting, as well as prospective stratification with DTI, MEP, reflex, and EMG measures, receives a higher priority. Tier 3 goes further to ecosystem delivery, stimulation-off retention and real-world function, workflow-ready service paradigms, reimbursement and equity-aware access, and governance of adaptive and AI-assisted control to determine scalable clinical effect.

A scalable ecosystem will need standards in place as opposed to offering technology. Interoperability (shared time bases, common triggers, and auditable logs) should be the priority of platforms to allow SCS, sensors, and robotic interfaces to be controlled in one common control scheme. Adaptive components must also be left under the control of a clinician, including fixed safety envelopes and explicit actions to take once signal loss or uncertainty occurs. To ensure that the stimulation parameters, as well as training dosage, are reproducible across sites, the protocols should delineate the stimulation parameters and training dosage, and patient stratification should identify who is likely to benefit enough to justify resource-intensive integration. Conclusively, the worth of multimodal neuromodulation will be identified by its ability to achieve retention when the stimulation is off and to increase functional participation in the real world, and not only because of performance during stimulation (Figure 3).

### 5.4. Clinical Translation Pathways: Trials, Regulation, and Health-System Integration

Bringing motor-restorative spinal cord stimulation (SCS) into a daily clinical routine requires a gradual cycle of evidence production, unlike the individual feasibility studies. The currently available data are to a large extent related to laboratory procedures carried out by very specialised teams [6]. In contrast to chronic neuropathic pain—where randomised controlled trials and longer-term follow-up studies have established a defined therapeutic role—motor-restorative applications in spinal cord injury and stroke remain primarily supported by small prospective cohorts and proof-of-concept investigations. The next logical move will be multicentre studies that standardise not only stimulation montages but also rehabilitation dose, and that prespecify outcomes under stimulation-on and stimulation-off conditions to separate the effect of facilitation and retention. Effectiveness in actual service constraints such as issues from staffing variability, heterogeneity of patients, and follow-up capacity should then in turn be assessed with pragmatic study designs.

Regulatory acceptance is another form of bottleneck because the current systems are based on analgesic indications. The protocols involving motor use might use different amplitudes, temporal profiles, and training-coupled modalities of delivery; thus, safety assessment must extend beyond the conventional pain endpoints to autonomic effects, longer-term changes in excitability, and device durability. Adaptive and closed-loop systems raise additional requirements: regulators will expect explicit safety envelopes, validated triggers for parameter change, and failure-mode analyses that specify how the system behaves during signal loss, motion artefacts, or unstable physiology. If machine learning components are used for optimisation or intent decoding, deployment also depends on auditable data provenance, cybersecurity protections, and clinician-in-the-loop governance [96].

Even with efficacy and clearance, implementation depends on health-system readiness. Motor-restorative programmes are resource-intensive because they couple implantation and programming to repeated, high-dose rehabilitation and often require coordinated roles across surgery, neurology, therapy, and engineering support. Workflow burdens—iterative tuning, frequent reassessment, and sustained training—do not map cleanly onto current reimbursement models, and high device and staffing costs risk concentrating access in a small number of centres. Without explicit service models, new indications could widen, rather than narrow, disparities in access to advanced neurorehabilitation. Without careful service design, there is a risk that investigational motor applications could expand technological capability without proportionate gains in equitable access.

A viable translational package therefore needs standardisation and infrastructure. Minimum requirements include harmonised reporting of stimulation parameters and rehabilitation dose, multidimensional outcomes that capture participation and real-world function, and biomarker-informed stratification to prioritise patients with residual pathway integrity. Equally important is the explicit distinction between stimulation-enabled performance and stimulation-independent retention in trial design and reporting, as the latter will ultimately determine whether motor-restorative SCS evolves from experimental neuromodulation to reproducible therapy. Multicentre trial networks can then support protocol iteration, safety surveillance, and evidence generation for regulators and payers. In practice, success will be judged not by laboratory performance, but by reproducible stimulation-off retention and feasible delivery across diverse clinical settings.

### 5.5. Ethical, Equity, and Access Considerations in Next-Generation Neuromodulation

As the use of spinal cord stimulation (SCS) for pain treatment evolves into that for motor recovery, the sphere of ethical concerns shifts to include the perioperative risk as well as the burden in the long term, the uncertainty, and expectation management. Informed consent discussions should be clear enough to make the distinction between performance under continued stimulation and recovery without continued stimulation, as the two have contrasting implications for patient autonomy and permanence. Patients must be given a realistic assessment of the hidden dose of the therapy, frequent reprogramming, high-intensity exercise, travelling requirements, and the possibility that functional changes will be conditional based on continued stimulation and not maintained post-withdrawal. With the use of adaptive or algorithmic control, consent should also specify what is automated, what aspects will be affected by the clinician, and how a patient may interrupt, disable, or discontinue the intervention.

Equity is not a secondary consideration and/or a downstream consideration: it is active in influencing access to restorative neuromodulation. Most of the multimodal protocols, such as SCS with robotics, sensor arrays, or cortical stimulation, can only be implemented in high-resource centres, meaning that geographic location and institutional infrastructure can override clinical indications. Rural-based patients, those with limited financial adaptability, or those who are out of organised neurorehabilitation systems, are faced with increased roadblocks such as travel requirements, work absenteeism, caregiver availability, and a local lack of follow-up capacity. Technological advancement may widen the inequities in the absence of clearly defined service models and mechanisms of reimbursement in the sense that it concentrates the benefits in a few specialised programmes.

Other barriers to entry are attributable to the complexity of the workflow. The deployment is often successful only when there are coordinated efforts by the surgeons, rehabilitation clinicians, engineers, and data experts, as well as dependable hardware support and prolonged longitudinal follow-ups. These requirements increase the workload and stress on the system, including the clinic schedule, employee workload, and equipment maintenance, and impose an increased patient load, in terms of the frequency of sessions, recalibration of equipment, and follow-up. These requirements are poorly supported in disjointed coverage regimes in motor-restorative indications. Practical scalability would, therefore, be greatly reliant on capacity-building endeavours such as special training, regional referral networks, and protocol engineering to reduce the setup time and staffing levels, as opposed to depending on the performance of the devices themselves.

Governance and efficacy should also be factored equally in the ethical assessment of responsible innovation in this field. Adaptive systems are supposed to work within safety envelopes that have been previously defined, provide clinician-in-the-loop control, and produce auditable logs so that they can reproduce the device behaviour under uncertain conditions. In physiology-controlled closed-loop systems and machine learning-controlled optimisation, data governance has become a central focus; hence, signal provenance, secure storage, access, and cybersecurity are now considered clinical safety requirements rather than peripheral technical aspects. A fair implementation plan would need the involvement of networked care models, with regional centres serving to support peripheral ones in suitable situations, remote monitoring where appropriate, and tiered provision channels to maintain core safety and effectiveness as well as addressing dependence on limited infrastructure.

## 6. Synthesis and Field-Level Perspective

Spinal cord stimulation (SCS) remains an established therapy for selected patients with chronic neuropathic pain. In recent years, its potential role has expanded toward motor restoration in spinal cord injury and stroke. Early studies suggest that SCS can modulate spinal circuits toward a more permissive functional state, particularly when stimulation is combined with structured, task-specific rehabilitation. However, evidence of durable, stimulation-independent motor recovery remains limited and heterogeneous across studies [97]. At present, motor-restorative applications should therefore be regarded as investigational rather than established therapy.

Future research should focus on clarifying the mechanisms, refining patient selection, and defining realistic clinical endpoints. Mechanistically, studies should distinguish between stimulation-dependent facilitation and longer-term adaptive change, and relate stimulation parameters to identifiable circuit recruitment patterns rather than functional outcomes alone. Technologically, robustness should be assessed under real-world conditions, including changes in posture, fatigue, and everyday movement. Adaptive systems must operate within clearly defined safety limits and transparent control frameworks.

Several challenges remain. Patient stratification is still imprecise, and validated biomarkers capable of predicting responses are lacking in populations with heterogeneous lesions and comorbidities. The reporting of stimulation parameters and rehabilitation dose varies across studies, making cross-protocol comparisons difficult. Multicentre trials with adequate statistical power and long-term follow-up are still scarce. At the same time, adaptive and machine learning–assisted systems are advancing rapidly, sometimes faster than the regulatory, safety, and governance structures required to support them. Resource demands remain high, and access is often limited to specialised centres.

A realistic path forward includes harmonised reporting standards, prospective stratification based on markers of residual pathway integrity, and trial designs that prespecify both stimulation-on and stimulation-off outcomes. For adaptive systems, clinician oversight and predefined safety boundaries should be integral components of system design. Importantly, translation should be judged not only by laboratory performance but also by reproducible stimulation-off retention and feasible implementation across diverse clinical settings. When these conditions are met, SCS may evolve from a promising neuromodulatory approach to a reproducible therapeutic option for defined patient groups, without overstating the current level of evidence.

## Figures and Tables

**Table 1:** Clinical indications, stimulation modes, and functional outcomes of spinal cord stimulation (SCS) across neurological disorders.

Condition	Indication Type	Stimulation Mode	Reported Clinical Outcomes	Effectiveness Estimate
Failed Back Surgery Syndrome (FBSS)	Chronic neuropathic pain	Tonic SCS, high-frequency (10 kHz) SCS	Sustained pain relief, improved physical function, reduced opioid use	50–70% achieve ≥50% pain reduction
Complex Regional Pain Syndrome (CRPS)	Refractory limb pain	Burst SCS, DTM-SCS	Decreased allodynia/hyperalgesia, functional improvement	~40–60% responder rate
Spinal Cord Injury (SCI)	Motor control restoration	Epidural SCS (eSCS), closed-loop SCS	Re-initiation of volitional stepping, trunk balance recovery	Recovered motor function in selected subacute SCI patients
Post-Stroke Hemiparesis	Gait and strength recovery	Cervical SCS + rehabilitative training	Increased grip strength, gait symmetry, proximal limb activation	Initial pilot results encouraging
Parkinson’s Disease	Freezing of gait, axial symptoms	Thoracic SCS (low-frequency burst)	Reduced freezing episodes, enhanced gait rhythmicity	Heterogeneous results; benefits in select cases
Multiple Sclerosis (MS)	Spasticity, sensorimotor dysfunction	Cervical/thoracic SCS (experimental)	Reduced tone, improved coordination (preliminary)	Limited human data, ongoing trials

**Table 2:** Major clinical case series and representative trials of spinal cord stimulation (SCS) across pain and motor-restorative indications.

Clinical Indication	Representative Study/Programme	Study Design	Sample Size	Key Reported Outcomes	Stimulation-Off Retention	Evidence Status
Chronic Neuropathic Pain (FBSS, CRPS, Radiculopathy)	Multiple multicentre RCTs (e.g., 10 kHz SCS trials; conventional tonic SCS trials)	Randomised controlled trials	*n* = 100–200+	≥50% pain reduction in ~50–70% of responders; improved quality of life; reduced opioid use in subsets	Sustained benefit at 6–12 months in many patients; long-term attrition reported	Established indication (RCT-supported)
Painful Diabetic Neuropathy	10 kHz high-frequency SCS trials	Randomised controlled trial	*n* ≈ 200	Significant pain reduction vs. medical management; improved sleep and function	Sustained at 12 months in responders	Established/expanding
Spinal Cord Injury (Motor Restoration—Epidural SCS)	Harkema et al. [7]; Angeli et al. [15]; selected centre-based programmes	Small prospective case series	*n* = 4–10 per cohort	Re-emergence of voluntary stepping; improved trunk control; stimulation-enabled standing	Frequently stimulation-contingent; limited consistent off-stimulation retention	Investigational (proof-of-concept)
Spinal Cord Injury (Transcutaneous SCS)	Pilot feasibility studies; single-centre programmes	Pilot trials/small cohorts	*n* = 5–20	Improved stepping-like activity; enhanced EMG recruitment patterns	Often diminished after stimulation withdrawal	Early-stage/exploratory
Post-Stroke Hemiparesis (Cervical SCS)	Powell et al. [89], Nature Medicine 2023	Small uncontrolled cohort	*n* = 2–3	Increased grip strength; improved reach-to-grasp; enhanced voluntary activation	Largely stimulation-dependent; durability under investigation	Early translational
Parkinson’s Disease (Gait/Freezing)	Small case series	Observational	*n* < 20	Reduced freezing episodes in selected patients	Inconsistent; heterogeneous response	Exploratory
Multiple Sclerosis/Other Neurological Disorders	Preliminary case reports	Case series	*n* < 10	Reduced spasticity; modest coordination gains	Insufficient data	Preliminary/hypothesis-generating

**Table 3:** Technological advancements and multimodal co-interventions enhancing spinal cord stimulation (SCS) for functional restoration.

Innovation/Co-Intervention	Mechanistic Contribution	Functional Implication
Closed-loop SCS	Real-time biofeedback; adaptive waveform tuning	Enhances motor output precision; stabilises long-term effects
BCI-SCS integration	Decodes volitional cortical intent to trigger spinal drive	Enables intentional movement in SCI patients
Peripheral stimulation pairing	Amplifies afferent feedback; promotes synaptic plasticity	Improves limb coordination, gait retraining
tDCS/tACS co-application	Modulates cortical excitability and gain control	Enhances responsiveness to SCS and motor relearning
Pharmacological priming (e.g., serotonergic)	Facilitates circuit recruitment and neuromodulatory tone	Supports motor pattern generation and plasticity
Intelligent parameter optimisation (AI)	Learns individual response profiles; predicts effective protocols	Enables personalised therapy scheduling and titration

## Data Availability

No new data were created or analysed in this study. Data sharing is not applicable to this article.

## Abbreviations

The following abbreviations are used in this manuscript:

AIS	American Spinal Injury Association Impairment Scale
CRPS	Complex Regional Pain Syndrome
ECAP	Evoked Compound Action Potential
EMG	Electromyography
FBSS	Failed Back Surgery Syndrome
FES	Functional Electrical Stimulation
ICF	International Classification of Functioning, Disability, and Health
PNS	Peripheral Nerve Stimulation
SCS	Spinal Cord Stimulation
SCI	Spinal Cord Injury
tDCS	Transcranial Direct-Current Stimulation
TMS	Transcranial Direct-Current Stimulation
VAS	Visual Analogue Scale
NRS	Numeric Rating Scale
ODI	Oswestry Disability Index
PGIC	Patient Global Impression of Change
